# Measurement of the Energy Intensity of Human Well-Being and Spatial Econometric Analysis of Its Influencing Factors

**DOI:** 10.3390/ijerph17010357

**Published:** 2020-01-05

**Authors:** Ruyin Long, Qin Zhang, Hong Chen, Meifen Wu, Qianwen Li

**Affiliations:** 1 School of Management, China University of Mining and Technology, Xuzhou 221116, China; longruyin@cumt.edu.cn (R.L.); rickkavence@cumt.edu.cn (Q.Z.); wumeifen@cumt.edu.cn (M.W.); 2 Research Center for Energy Economics, School of Business Administration, Henan Polytechnic University, Jiaozuo 454000, China

**Keywords:** energy intensity of human well-being, influencing factors, spatial econometric analysis, China

## Abstract

Current energy efficiency indicators (such as energy intensity) do not properly reflect the inherent relationship between “energy-environment-health”. Therefore, this study introduces the indicator of energy intensity of human well-being (EIWB) to depict the efficiency problem between energy consumption and residents’ health. In this paper, panel data of 30 provinces in mainland China from 2005 to 2016 is used to calculate the EIWB of each province and analyze its spatial distribution. Moreover, the effect of influencing factors on EIWB is investigated by using the spatial Durbin model. The results show that: (1) The EIWB presents a spatial agglomeration. The provinces with high EIWB mostly cluster in the northern China. (2) Industrial structure and energy structure have positive effects on EIWB in local area through increasing energy consumption and damaging residents’ health. (3) The effect of urbanization and income on local EIWB is significantly positive because it will promote energy consumption. (4) Industrial structure, health expenditure, foreign direct investment and technological progress have spatial spillover effects due to its significant impact on residents’ health in neighboring areas. Based on conclusions, the corresponding policy recommendations are proposed.

## 1. Introduction

In recent years, the problem of environmental pollution has become more and more serious, and the frequent occurrence of air pollution such as haze and PM 2.5 has caused great threat to residents’ health [1]. Environmental pollution has become a hot issue concerned by the whole society, and the coal-based energy consumption and huge total energy consumption are the main causes of environmental deterioration. According to research, although energy consumption promotes economic development, it will have serious negative impacts on residents’ health conditions, including respiratory diseases, cancer and even lessen children’s cognitive ability [2,3,4]. Therefore, improving energy efficiency can be an important way to improve environmental quality and residents’ health and well-being.

There is a great quantity of literature on energy efficiency and the measurement of energy efficiency is the main concern. The most widely used indicator to measure energy efficiency is energy intensity (EI), which is measured by the ratio of the total energy consumption to the gross domestic product (GDP). This indicator reflects the relationship between energy consumption and economic activities. The lower the energy intensity, the higher the energy efficiency of economic development is. At the same time, reducing energy intensity can reflect the improvement of the environment to some extent. However, many scholars criticized the energy intensity as rough and inaccurate [5] because it only considers the relationship between energy input and economic output (usually GDP). As an improvement, researchers in related fields have proposed stochastic frontier analysis methods [6,7] and data envelopment methods [8,9] based on multiple input factors to achieve more accurate energy efficiency estimation. These studies result in an improved energy efficiency indicator: Total factor energy efficiency (TFEE). However, these studies still do not deviate from the thinking mode of measuring energy efficiency by energy input and economic output, and do not properly reflect the inherent relationship between “energy-environment-health”, ignoring the damage to human well-being caused by environmental pollution, which was led by energy consumption. In result, there is a lack of consideration for residents’ health and well-being in the formulation of energy efficiency goals. Therefore, the present study introduces a new energy efficiency indicator: Energy intensity of human well-being (EIWB). Idea of EIWB can be traced back to the related research on sustainability. Researchers in this field have questioned whether the growth of energy consumption can actually improve human well-being, and conducted a series of studies on this [10,11,12]. The environmental Kuznets curve (EKC) is one of the important research findings for this challenge. Based on this theory, Dietz et al. [13] defined the ecological intensity of well-being by the ratio of ecological footprint to the life expectancy (LE). Jorgenson et al. [14] first proposed and defined the EIWB according to Dietz’s research, and estimated the energy intensity of well-being (EIWB) by the energy consumption per capita (ECPC) to the life expectancy (LE). In Jorgenson’s study, life expectancy per capita exists as a proxy of health and well-being, so the definition of EIWB can also be extended to EIWB = ECPC/WB, where WB is well-being. This formula is exactly the basis for the estimation of EIWB in this paper. It can be seen from the calculation method that EIWB reflects the efficiency problem between energy consumption and residents’ well-being. Reducing EIWB means reducing energy consumption while maintaining or improving residents’ well-being.

Compared with the existing energy efficiency indicators, one of the main advantages of EIWB is that EIWB provides a new perspective of thinking and research. EI and TFEE both define energy efficiency as the relationship between energy input and economic output [15,16,17], while economic output cannot be simply equal to the health status and well-being of residents [18]. In other words, at the beginning of design, EI and TFEE did not highlight that the ultimate goal of economic development is to achieve development of human being. EIWB links energy consumption with residents’ health status and well-being, thus it can better reflect the impact of environmental damage that result from energy consumption on human health in a country or a region. Therefore, EIWB can provide more comprehensive information for sustainable development than EI and TFEE.

Another advantage is that EIWB is more concise and operable. TFEE argues that energy itself cannot result in economic output [19], so it measures energy efficiency considering multi input factors. Using improved estimation method, the accuracy of TFEE is greatly improved compared with EI, which makes TFEE a better choice in academic research on energy efficiency. However, in practice, TFEE is not simple and operable. Table 1 shows some researches on TFEE. It can be seen from Table 1 that there are many input factors to be considered in the calculation of TFEE and the calculation is complex. Therefore, it is very difficult for policy makers to formulate clear and unified energy planning policies for TFEE, which is also an important reason that the Chinese government takes energy consumption per unit of GDP (EI) as the goal of energy policy planning in the 12th and 13th Five-Year plans. Different from TFEE, EIWB only involves two variables, and the data of these two variables are very easy to obtain. Hence, EIWB is of superior simplicity and operability, which allows it to be used as a reference standard for regulators to formulate energy policies.

The main contributions of this study are: (1) in terms of indicator construction, this paper introduces the EIWB, which can examine the trade-off relationship between energy consumption and residents’ well-being in China from the perspective of energy efficiency, thus expanding the indicator system of energy efficiency. (2) In terms of indicator measurement methods, this paper uses population mortality, infant mortality and maternal mortality to measure EIWB of 30 provinces in China. This is different from previous research, which expands the measurement method of EIWB to a certain extent, and provides a solution for the measurement of indicators without average life expectancy data. (3) In terms of the evaluation system, EIWB can help evaluate the inherent relationship between “energy-environment-health” and provide a scientific basis for the government and industry to formulate a sustainable policy based on local conditions. It provides theoretical and empirical guidance for establishing cross-regional cooperation mechanisms in various regions and synergistically reducing EIWB.

## 2. Research Methodology

### 2.1. The Measurement of EIWB

This study proposed EIWB as a new indicator to explore energy efficiency. It is essential to appropriately measure EIWB before conducting further investigation. EIWB is the ratio of energy consumption per capita to health conditions. Therefore, the key to measure EIWB is how to evaluate residents’ health and well-being. Jorgenson et al. [14] used life expectancy per capita as the proxy of human health and quality of life in his research, but they also repeatedly mentioned that life expectancy per capita may not be the best index that can describe human life quality and health, and the important reason for selecting this indicator was its availability. At present, scholars at home and abroad mainly make use of the following two categories of indicators to evaluate the residents’ actual health conditions: (a) mortality indicators such as population mortality, infant mortality, under-five mortality, maternal mortality and (b) life expectancy indicators such as average life expectancy and disability adjusted life expectancy [27,28]. Since China’s average life expectancy, disability adjusted life expectancy and other life indicators in provincial level are only counted and published in the census year and a few other years, this study used population mortality, infant mortality and maternal mortality to comprehensively evaluate the residents’ well-being in each region. An entropy weight method [29] was employed to calculate a synthetical index of residents’ health and well-being using negative health indicators mentioned above. Then we normalized this synthetical index in order to make it a positive health indicator. This positive health indicator was used as WB in this study. EIWB was calculated by the ratio of the per capita energy intensity to WB, which was obtained by the method we discussed above.

Given that EIWB would be used as a dependent variable in further studies, an additional problem needs to be addressed here: since the variation coefficient of the numerator (energy consumption per capita) is smaller than that of the denominator (health indicator; note: the variation coefficient is the ratio of standard deviation to mean, reflecting the dispersion degree of data), that is, the change of health indicators has a greater impact on the overall index than the energy consumption per capita. Therefore, it is necessary to subtract a constant from the numerator to balance the variation coefficient of the numerator and the denominator. This method is widely used in the studies of EIWB [30,31]. The basic mathematical basis for this is that subtracting a constant from the numerator reduces the mean without changing the standard deviation of the numerator, thereby equalizing the variation coefficients in the numerator and denominator. In this study, the standard deviation of the numerator is 1502.868, and the mean value is 3214.246 (kg standard coal/ person). The standard deviation of the denominator is 7.522 and the mean value is 12.670. The calculation shows that when the constant 682.847 is subtracted from the numerator, the coefficient of variance between the numerator and the denominator can be balanced. Therefore, the calculation formula of EIWB in this study is:

(1) $$EIWB=\frac{ECPC-682.847}{WB}.$$

### 2.2. Spatial Characteristics of EIWB

The study first used GeoDa to draw spatial quartile maps of EIWB to visually analyze the spatial distribution of EIWB in China from 2005 to 2016. Then, in order to ensure its strictness and science, statistical methods are used to further confirm the spatial autocorrelation and agglomeration characteristics. Specifically, Moran’s I statistic was used as the evaluation standard. The Moran’s I was first proposed in 1950 by Pierce Moran, an Australian statistician. It can be used to describe the degree of association between spatial units: the positive value of Moran’s I indicates that there is spatial agglomeration, while the negative indicates that there is spatial diffusion. The larger the value of the Moran’s I, the higher the degree of spatial agglomeration or diffusion is. Moran’s I can be divided into global Moran’s I and local Moran’s I. We first employed global Moran’s I to figure out spatial autocorrelation characteristics of EIWB as a whole. However, the global Moran’s I can only describe the average degree of association between the various regions in the global range, but when there is positive spatial autocorrelation in some regions and negative spatial autocorrelation in other regions, mutual offset will occur. Therefore, it has certain limits in reflecting the degree of spatial correlation. Based on this, this study employed local Moran scatter plots to further examine the spatial autocorrelation characteristics of EIWB. The formulas of global Moran’s I statistic and local Moran’s I are as follows:

(2) $$Global\;Moran’s\;I=\;\frac{n\sum_{i=1}^{n}\sum_{j=1}^{n}W_{ij}(y_{i}-\bar{y})(y_{j}-\bar{y})}{(\sum_{i=1}^{n}\sum_{j=1}^{n}W_{ij})\sum_{i=1}^{n}{\left(y_{i}-\bar{y}\right)}^{2}},$$

(3) $$Local\;Moran’s\;I\;=\;\frac{n(y_{i}-\bar{y})\sum_{j=1}^{n}W_{ij}(y_{j}-\bar{y})}{\sum_{i=1}^{n}{\left(y_{i}-\bar{y}\right)}^{2}},$$

where $y_{i}$ and $y_{j}$ represent the EIWB of province *i* and province *j* respectively. *n* represents the number of regions, which is 30 in this study. $\bar{y}=\frac{1}{n}\sum_{i=1}^{n}y_{i}$ is the mean of the EIWB of all provinces. *W* is the spatial weight matrix. There are three kinds of spatial weight matrix, which are commonly used in related spatial econometrics research. They are spatial contiguity matrix, spatial distance matrix and spatial economy matrix and are respectively represented by *W*_1_, *W*_2_ and *W*_3_ in this study. Each of three matrices describes a kind of association among different regions. The spatial contiguity matrix is the most commonly used spatial weight matrix. In a spatial contiguity matrix, elements that are geographically adjacent are defined as 1 while those that are not geographically adjacent are defined as 0. The spatial contiguity matrix is usually a good choice for spatial analysis but it can be biased because of simplicity under particular circumstance. Thus, the spatial distance matrix adopts the inverse of the distance to enhance the robustness. However, both of two matrices only reflect the relationship of geographical location. Different from the spatial contiguity matrix and the spatial distance matrix, the spatial economic matrix can also reflect the economic relationship among different regions. To define a spatial economic matrix, GDP of different regions are usually used [32]. Specifically, the rules for determining each element in the spatial contiguity matrix *W*_1_, spatial distance matrix *W*_2_ and spatial economic matrix *W*_3_ are shown in (4), (5) and (6), respectively:

(4) $$w_{ij}=\{\begin{array}{cc} 1 & \mathrm{Geographically}\;\mathrm{adjacent}\\ 0 & \mathrm{Geographically}\;\mathrm{not}\;\mathrm{adjacent} \end{array}.$$

(5) $$w_{ij}=\{\begin{array}{cc} \frac{1}{distance_{ij}} & i\neq j\\ 0 & i=j \end{array}.$$

(6) $$w_{ij}=\{\begin{array}{cc} \frac{1}{\left|\bar{GDP_{i}}-\bar{GDP_{j}}\right|} & i\neq j\\ 0 & i=j \end{array}.$$

In this study, we adopted only spatial contiguity matrix to test if there is a spatial autocorrelation of EIWB in China due to limited space. The spatial distance matrix and spatial economic matrix will be adopted in empirical models to ensure robustness of estimation.

### 2.3. Spatial Econometric Analysis

We argued that EIWB is a better indicator to help government realize sustainability than traditional indicators, such as EI and TFEE. By reducing EIWB, the society can enhance its energy efficiency as well as residents’ well-being. In order to explore how to reduce EIWB in China, it is of importance to figure out the factors, which have significant influence on EIWB and whether they have a positive effect or a negative effect. Therefore, this study employed a spatial econometric model to analyze the impact of the influencing factors of EIWB.

#### 2.3.1. Variable Selection of Influencing Factors

Both EIWB and traditional energy intensity depict energy efficiency to some extent, but EIWB also reflects residents’ well-being. Therefore, when selecting the influencing factors of EIWB, the existing studies on energy intensity and human health and well-being are referred. Although part of these influencing factors had been studied in other energy efficiency researches, they have different influence paths in this study. They can influence EIWB not only by influencing energy consumption but also by influencing residents’ health, which makes our result and conclusion different.

Through reviewing large number of literature research, the main influencing factors are finally summarized as follows:

Industrial structure (IS). The industrial structure has always been considered as one of the influencing factors of energy intensity, but there are different opinions on how the industrial structure affects energy intensity. There are currently several opinions: First, the adjustment of industrial structure will have a greater impact on energy intensity and promote the reduction of energy intensity [33,34,35]. Second, the impact of industrial restructuring on energy intensity reduction is weak and may even have a negative impact [36,37]. Therefore, the influence of industrial structure on EIWB remains to be further explored. This paper describes the industrial structure by the proportion of industrial output value in each province.

Urbanization level (UL). Yan [38] believed that the improvement of urbanization level could significantly promote to the growth of energy consumption. Liu [39] found that there had unilateral Granger casual-effect relationship between urbanization level and energy consumption, and the impact of urbanization level on energy consumption has weakened with time. In this paper, the proportion of urban residents in the total population at the end of the year is adopted to depict the urbanization level of provinces.

Energy structure (ES). In terms of the energy structure, some scholars believed that its contribution to the change of energy intensity was low, because China’s rich resource endowment of coal restricted the adjustment of energy consumption structure [40]. However, EI Anshasy and Katsaiti [41] found that there is a long-term significant relationship between energy consumption structure and energy intensity. Through empirical analysis, Guo et al. [42] found that the proportion of coal in energy consumption and energy intensity have a positive effect. This paper also uses the proportion of coal in energy consumption to quantify the energy structure based on relevant study.

Foreign direct investment (FDI). Research on the impact of FDI on the environment suggests that FDI has two hypotheses for “pollution paradise” and “pollution halo” for environmental impacts in different regions. “Pollution paradise” refers to the area where pollution-intensive industries will be more relaxed with the inflow of FDI into the environment, making the inflow area gradually become a “pollution paradise”. “Pollution halo” believes that FDI will bring advanced production technologies into the region, improve the energy efficiency of the region and play a positive role in improving the environment. It is generally believed that the impact of FDI on energy efficiency varies according to regional differences [43].

Per capita disposable income (PCDI). The increase of income level usually leads to the increase of energy consumption, and many scholars have also studied the relationship between this factor and energy intensity [44,45]. Meanwhile, income level can also affect individual health status [46,47], so this factor should be considered as one of the important factors affecting EIWB.

Public health expenditure (HE). Public health expenditure reflects the medical and health level of the region to some extent, which affects the EIWB by influencing the health level of residents [48,49].

Technological progress (T). Technological progress has always been considered as one of the major factors contributing to the decline of energy intensity [50]. Through a quantitative study of panel data from 30 provinces in China from 2000 to 2013, Huang et al. [51] found that technical factors have a significant positive effect on the reduction of energy intensity. In this paper, the number of patents granted at the end of each year in all provinces is used to describe the technological progress.

#### 2.3.2. Model Setting

The initial definition of EIWB is consistent with the environmental Kuznets curve (EKC), that is, dependent variables are non-expected outputs. Since energy consumption is the main source of many pollutant emissions, energy consumption can be considered as a source of total pollutants. Based on this logic, the model can be initially set up by using the STIRPAT model commonly used in EKC research:

(7) $$\begin{array}{ll} \ln eiwb_{it}= & \ln a_{it}+\beta_{1}\ln is_{it}+\beta_{2}\ln ul_{it}+\beta_{3}\ln es_{it}+\\ & \beta_{4}\ln fdi_{it}+\beta_{5}\ln pcdi_{it}+\beta_{6}\ln he_{it}+\beta_{7}\ln t_{it}+\ln\varepsilon_{it} \end{array}$$

In Model (7), *eiwb*, *is*, *ul*, *es*, *fdi*, *pcdi*, *he* and *t* respectively represent energy intensity of human well-being, industrial structure, urbanization level, energy structure, foreign direct investment, per capita disposable income, public health expenditure and technological progress.

Considering that the EIWB and its influencing factors have geospatial effects, a spatial econometric model can be constructed based on the STIRPAT model. The reason why we chose the spatial econometric method was: the previous investigation confirmed that there is a spatial autocorrelation of EIWB in China, which obviously violates the classic assumption that variables should be independent of each other. Under such circumstance, spatial econometric methods tend to perform better than other classic econometrics methods. A great quantity of literature has affirmed the argument [52,53].

Elhorst [54] pointed out that the spatial lag model (SAR) and the spatial error model (SEM) were special forms of the spatial Durbin model (SDM). In the form of spatial model, the spatial Durbin model containing both dependent and independent lag terms should be preferred. After various tests, it is judged whether the model needs to be transformed into a spatial lag model or a spatial error model. Therefore, the following model is established:

(8) $$\begin{array}{ll} \ln eiWb_{it}= & \alpha +\rho W\ln eiwb_{it}+\beta_{1}\ln is_{it}+\beta_{2}\ln ul_{it}+\beta_{3}\ln es_{it}+\beta_{4}\ln fdi_{it}\\ & +\beta_{5}\ln pcdi_{it}+\beta_{6}\ln he_{it}+\beta_{7}\ln t_{it}+\theta_{1}W\ln is_{it}+\theta_{2}W\ln ul_{it}+\theta_{3}W\ln es_{it}\\ & +\theta_{4}W\ln fdi_{it}+\theta_{5}W\ln pcdi_{it}+\theta_{6}W\ln he_{it}+\theta_{7}W\ln t_{it}+\mu_{it}+\varepsilon_{it} \end{array}$$

In Model (8), W is a 30 × 30 distance-based adjacent geographic matrix. $W\ln eiwb_{it}$, $W\ln is_{it}$, $W\ln ul_{it}$, $W\ln es_{it}$, $W\ln fdi_{it}$, $W\ln pcdi_{it}$, $W\ln he_{it}$ and $W\ln t_{it}$ are the spatial lags of EIWB, industrial structure, urbanization level, energy structure, foreign direct investment, per capita disposable income, public health expenditure and technological progress respectively. $\mu_{it}$ is the individual fixed effect. $\varepsilon_{it}$ is the random error term. The meanings of the remaining variables are consistent with those in Model (7).

Next, the Wald test and LR test (likelihood ratio test) were used to determine whether the spatial Durbin model could be transformed into a spatial lag model or a spatial error model. In Table 2, the results of Wald test and LR test were both significant at the significant level of 1%, so the null hypothesis was rejected, and it is considered that the spatial Durbin model cannot be simplified into a spatial error model or a spatial lag model. Since the Hausman test results were significant, the spatial Durbin model with fixed effects was finally selected for estimation.

### 2.4. Decomposition of Spatial Effects

LeSage [55] proposed that the total effect of explanatory variables in spatial Durbin model could be decomposed into direct effect and indirect effect by means of partial differential decomposition. Among them, the direct effect refers to the influence of local factors on local region, while the indirect effect refers to the influence of local factors on adjacent regions.

Supposing a simplified SDM model can be expressed as:

(9) y= α+ρWy+Xβ+WXθ+ε.

Denoting

(10) $$X\beta \;=\;\left(x_{1},x_{2},\cdots ,x_{k}\right){\left(\beta_{1},\beta_{2},\cdots ,\beta_{k}\right)}^{T}=\sum_{i=1}^{k}x_{i}\beta_{i}.$$

(11) $$X\theta \;=\;\left(x_{1},x_{2},\cdots ,x_{k}\right){\left(\theta_{1},\theta_{2},\cdots ,\theta_{k}\right)}^{T}=\sum_{i=1}^{k}x_{i}\theta_{i}.$$

(12) $$A={\left(I_{n}-\rho W\right)}^{-1}=I_{n}+\rho W+\rho^{2}W^{2}+\rho^{3}W^{3}+\cdots .$$

(13) $$B_{s}=A(I_{n}\beta_{s}+W\theta_{s}).$$

Then, Equation (9) can be rewritten as Equation (14):

(14) $$y=\left(\begin{array}{c} y_{1}\\ y_{2}\\ \vdots \\ y_{n} \end{array}\right)=\sum_{s=1}^{k}B_{s}x_{s}+A\alpha +A\varepsilon =\sum_{s=1}^{k}\left(\begin{array}{cccc} \frac{\partial y_{1}}{\partial x_{1s}} & \frac{\partial y_{1}}{\partial x_{2s}} & \cdots & \frac{\partial y_{1}}{\partial x_{ns}}\\ \frac{\partial y_{2}}{\partial x_{1s}} & \frac{\partial y_{2}}{\partial x_{2s}} & \cdots & \frac{\partial y_{2}}{\partial x_{ns}}\\ \vdots & \vdots & \ddots & \vdots \\ \frac{\partial y_{n}}{\partial x_{1s}} & \frac{\partial y_{n}}{\partial x_{2s}} & \cdots & \frac{\partial y_{n}}{\partial x_{ns}} \end{array}\right)\left(\begin{array}{c} x_{1s}\\ x_{2s}\\ \vdots \\ x_{ns} \end{array}\right)+A\alpha +A\varepsilon .$$

Thus, the direct effect, the total effect and the indirect effect of variable $x_{s}$ can be respectively calculated by Formulas (15)–(17):

(15) $$Direct\;effect=\frac{1}{n}tr(B_{s}).$$

(16) $$Total\;effect=\frac{1}{n}\sum_{i=1}^{n}\sum_{j=1}^{n}B_{s}.$$

(17) $$Indirect\;effect=Total\;effect-Direct\;effect=\frac{1}{n}\left[\sum_{i=1}^{n}\sum_{j=1}^{n}B_{s}-tr(B_{s})\right].$$

### 2.5. Data Collection

This study first measured EIWB of China’s provincial administrative regions (excluding Tibet, Hong Kong, Macau and Taiwan) from 2005 to 2016. The energy consumption per capita data of each region came from China Energy Statistical Yearbook and China Statistical Yearbook. The data of population mortality, infant mortality and maternal mortality were from China Health Statistics Yearbook and National Maternal and Child Health Monitoring.

Then, a spatial econometric analysis was employed. The empirical sample of this part was the balanced panel data formed by China’s 30 provincial administrative regions (excluding Tibet, Hong Kong, Macao and Taiwan) from 2005 to 2016. Data of industrial structure, urbanization level, foreign direct investment, per capita disposable income and quantity of patent authorization at the end of each year were from the China Statistical Yearbook, which is supplemented by the statistical yearbook of each province. Energy structure data was sorted out based on the China Energy Statistics Yearbook. Public health expenditure data was from the China Finance Yearbook.

## 3. Results and Discussion

### 3.1. Values of EIWB and Its Distribution

**Figure 1:**
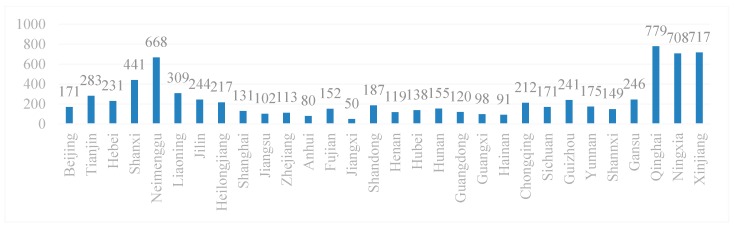
Annual average values of energy intensity of human well-being (EIWB) in each region.

**Figure 2:**
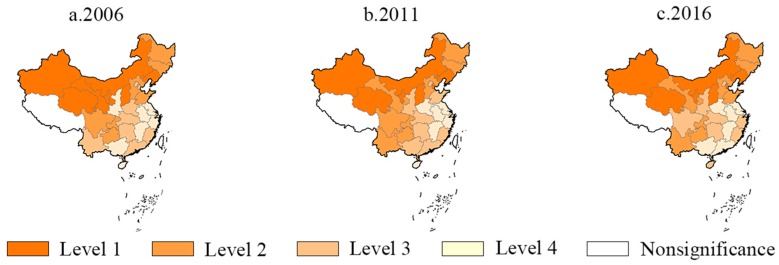
(**a**) Spatial distribution quartile maps of EIWB in 2006; (**b**) spatial distribution quartile maps of EIWB in 2011 and (**c**) spatial distribution quartile maps of EIWB in 2016.

Using the method mentioned in research methodology to measure EIWB, the annual average values of EIWB in each region from 2005 to 2016 are shown in Figure 1:

GeoDa was used to draw spatial quartile maps of EIWB to visually analyze the spatial distribution of EIWB in China from 2005 to 2016. Due to limited space, the spatial distribution of EIWB in 2006, 2011 and 2016 is presented at 5-year intervals to ensure simplicity. In Figure 2a–c are the spatial quartile maps of China’s EIWB in 2006, 2011 and 2016 respectively. 

In spatial distribution maps above, shades of different regions reflect its grade of EIWB respectively. Darker color in a province indicates a higher grade of EIWB. In particular, blank areas are provinces with missing data. Except the blank areas, the provinces in China can be divided into four grades according to the color from dark to light. The first grade contains seven provinces with the highest EIWB in China, which are shown with the darkest colors in the figure. The second and third grades each include eight provinces. The remaining seven of the lightest areas are at the fourth grade.

The spatial quartile maps show the agglomeration and heterogeneity of spatial distribution of EIWB. On the whole, the agglomeration of China’s EIWB was very obvious. The provinces with high EIWB mainly clustered in northern China, while the provinces with low EIWB were mainly concentrated in the southeast coastal areas. In 2006, the seven provinces with the highest EIWB were Liaoning, Inner Mongolia, Shanxi, Ningxia, Gansu, Qinghai and Xinjiang. In 2011 and 2016, the seven provinces with the highest EIWB were Tianjin, Liaoning and Inner Mongolia, Shanxi, Ningxia, Qinghai and Xinjiang. Comparing the spatial distribution of EIWB in these three years it could be found that EIWB of Liaoning, Inner Mongolia, Shanxi, Ningxia, Qinghai and Xinjiang were high, and Gansu’s EIWB fell from the first grade to the second grade during the inspection period. While Tianjin’s EIWB increased, becoming one of the new provinces with the highest grade of EIWB.

Spatial distribution maps suggest that there might be a strong spatial autocorrelation of EIWB between all provinces in China, and the provinces with high EIWB mainly clustered in the northern part of China. Specially, Liaoning, Inner Mongolia, Shanxi, Ningxia, Qinghai or Xinjiang had high EIWB during the study period. The reason for this phenomenon might be that the per capita energy consumption was too high or the health and well-being of the residents was poor, or both, these three aspects will be discussed in detail in the following.

**Figure 3:**
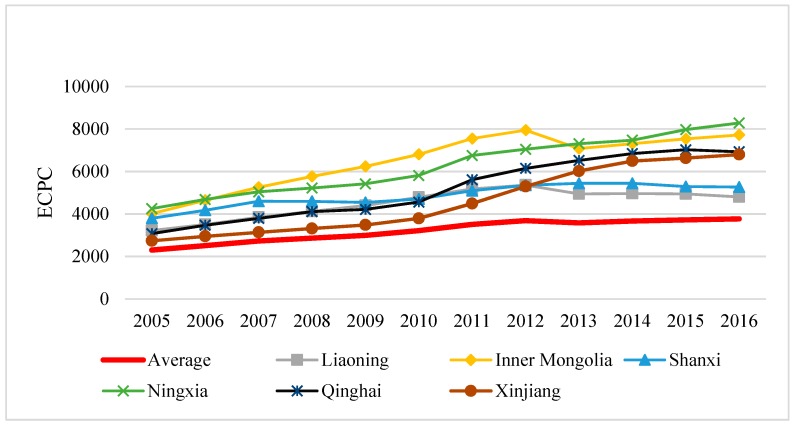
Per capita energy consumption (ECPC) of high EIWB provinces in 2005–2016.

In terms of energy consumption per capita (ECPC), it is confirmed from Figure 3 that ECPC of six provinces were higher than the national average. Among them, the growth rate of ECPC in Ningxia, Qinghai and Xinjiang was significantly higher than the national average, which might result from these provinces being exceptionally rich in petrochemical resources, and energy production is a pillar industry in these provinces. Therefore, ECPC of these provinces was affected to some extent, which was consistent with the research conclusions of Yan et al. and Wu et al. [56,57].

**Figure 4:**
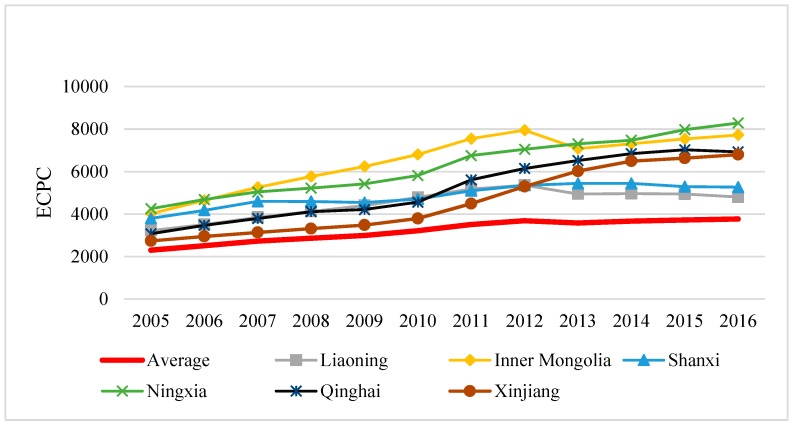
Health and well-being of residents with high EIWB from 2005 to 2016.

In terms of residents’ health and well-being, Figure 4 shows that except Liaoning, other provinces were lower than the national average, which might be due to the environmental damage caused by excessive dependence on resource-based industries. Wang et al. [58] supported this inference, and found that the richness of resources in a province was inversely related to its emission efficiency, and the provinces with richer resources have lower carbon emission efficiency and greater the damage to the environment and human well-being.

**Figure 5:**
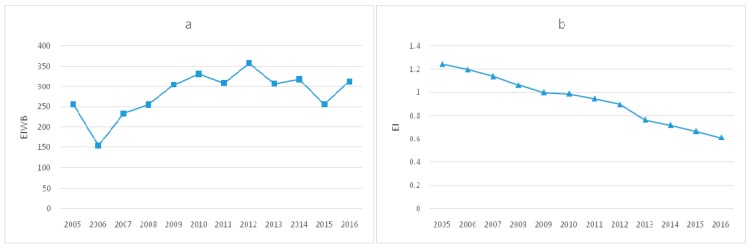
(**a**) Trends of EIWB in Tianjin and (**b**) trends of EI (energy intensity) in Tianjin.

In addition, this study also noticed a special case, Tianjin. In order to visually show the differences in EIWB and EI in assessing Tianjin’s energy efficiency, (a) and (b) in Figure 5 respectively presents the trends of Tianjin’s EIWB and EI over time. As can be seen from the Figure 5, different from the steady decline in EI, EIWB appeared as a larger fluctuation, and observations in many years show an increase over the previous year. From Figure 5a above, it can be seen that the grade of EIWB in Tianjin rose to the highest in 2016, reflecting the decline of energy efficiency. That was contrary to the research conclusions of Zhao and Lu [59] on the increase of energy efficiency in Tianjin, which was consistent with the decrease of EI shown in Figure 5b.

**Figure 6:**
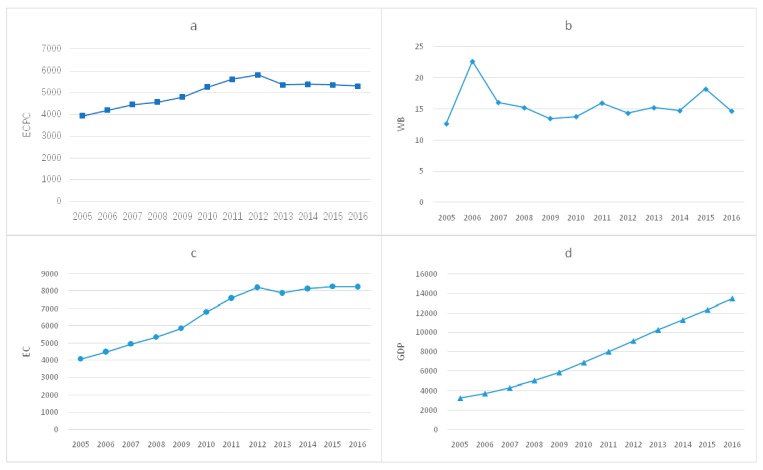
(**a**) Trends of ECPC in Tianjin; (**b**) trends of WB in Tianjin; (**c**) trends of EC (energy consumption) in Tianjin and (**d**) trends of GDP in Tianjin.

In order to explain the significant difference between the two energy efficiency indicators in evaluating Tianjin’s energy efficiency, this study further explored the trends of ECPC, WB, EC and GDP in Tianjin. It can be seen from Figure 6 that ECPC and EC in Tianjin is basically the same, showing a trend of going up with a continuously decreasing speed. However, during 2005–2016, the health and well-being of residents in Tianjin had not been improved significantly, there even were several obvious drops instead, resulting in the rise of EIWB in Tianjin. Different from the fluctuation of residents’ health and well-being, Tianjin’s real GDP increased steadily year by year, and the growth rate was obviously faster than EC, which was the main reason for the decrease of EI. The case of Tianjin shows that economic growth did not necessarily mean the improvement of well-being, and the assessment of energy efficiency based on EIWB could better reflect the internal connection of “energy-environment-health”.

### 3.2. Spatial Autocorrelation of EIWB

The global Moran’s I statistic of EIWB in China’s provinces from 2005 to 2016 was calculated by Stata 15.0. The results are shown in Table 3. It could be found that from 2005 to 2016, the global Moran’s I index of EIWB was significantly positive at significant level of 1%, the Moran’s I statistic of each year fluctuated between 0.354 and 0.409, indicating that the EIWB was in agglomeration.

**Figure 7:**
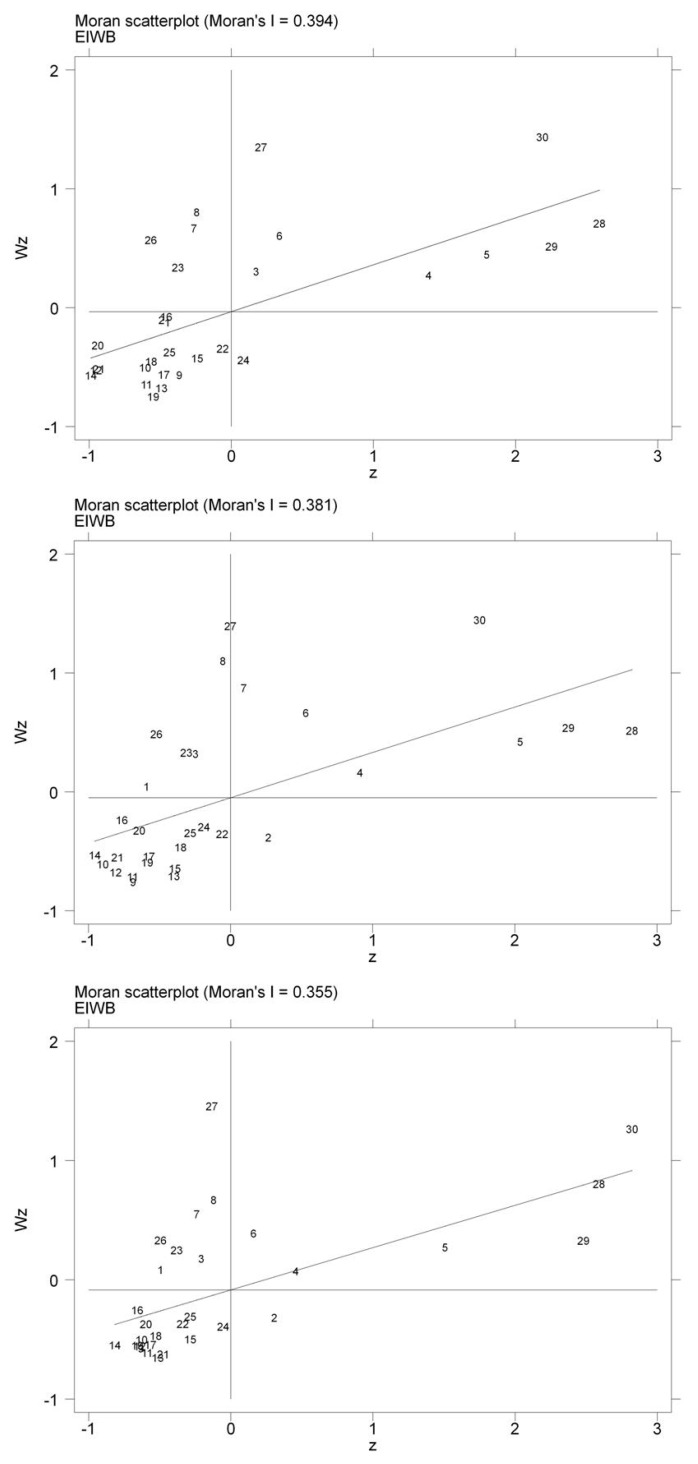
Local Moran scatter plots of China’s EIWB (in 2006, 2011 and 2016). Note: The numbers in the figure represent provinces, and their position in each quadrant was compiled based on the calculation results of Local Moran’s I.

Local Moran’s I was also calculated to draw the Moran scatter plots to confirm the conclusion that EIWB in China showed a spatial autocorrelation. The Moran scatter plots in Figure 7 respectively show the cluster types of EIWB in China. Among them, the horizontal axis represents EIWB, and the vertical axis represents the spatial lag of EIWB. There were four quadrants in a scatter plot that started at the top right and rotate counterclockwise. The four quadrants were: the first quadrant was the high–high (H–H) agglomeration quadrant, the scatter point in the quadrant indicates that the observations of EIWB in both local and neighboring region were relatively high. The second quadrant was a low–high (L–H) agglomeration quadrant, the scatter points in the quadrant indicate that the observations in the local region were relatively low, while the observations in the neighboring region were relatively high. The third quadrant was a low–low (L–L) agglomeration quadrant, the scatter point in the quadrant indicates that the observations of EIWB in both local and neighboring region were relatively low. The fourth quadrant was a high–low (H–L) agglomeration quadrant, the scatter points in this quadrant indicate that the observations in the local region were relatively high and the observations in the neighboring region were relatively low.

It can be seen from Figure 7 and Table 4 that, in 2006, there were 25 provinces in the first quadrant (H–H) and the third quadrant (L–L), accounting for 83.33% of the total provinces. In 2011, they were 23 provinces located in the first and third quadrants, accounting for 76.67% of the total provinces. In 2016, there were 22 provinces in the first and third quadrants, accounting for 73.33% of the total provinces. This indicates that the conclusions about the spatial agglomeration of EIWB obtained from the global Moran’s I index were correct. Comparing the number of scatter points in the first and third quadrant, there were eight scatter points (also known as “hot spots”) in the first quadrant and 17 scatter points (also known as “cold spots”) in the third quadrant in 2006. In 2011, there were seven “hot spots” and 16 “cold spots”. In 2016, there were six “hot spots” and 16 “cold spots”. Obviously, the number of "cold spots" was much higher than that of “hot spots”, which indicates that cluster type of EIWB in China was mostly low–low agglomeration.

### 3.3. Spatial Econometric Analysis of the Influencing Factors of EIWB

Three kinds of spatial matrix were adopted to ensure the robustness of estimation. In Table 5, the empirical results obtained by respectively using spatial contiguity matrix *W*_1_, spatial distance matrix *W*_2_ and spatial economic matrix *W*_3_ are presented. It can be seen from Table 5 that all three SDM models present basically consistent results on how each factor influenced EIWB. Specifically, the coefficients of industrial structure, urbanization level and energy structure were all significantly positive in all three models, indicating that the rise of these factors can result in an increase of EIWB. While coefficients of FDI and technical progress were negative at 1% significant level, suggesting that encouraging FDI and technical progress could effectively reduce EIWB. In addition, the coefficients of per capita disposable income in SDM models based on *W*_1_ and *W*_2_ were significantly positive while the coefficient estimated in the model based on *W*_3_ was not significant. This might indicate that the increase of per capita disposable income had a positive influence on EIWB in neighboring or geographically close regions but did not have a significant influence on economically similar regions. Similarly, it can be seen that public health expenditure could negatively influence EIWB only in neighboring regions. It can also be seen that all spatial lag coefficients obtained by using different model based on three spatial weight matrices, which were significant, remained consistent.

According to the research of LeSage [55], it will cause a bias if using the coefficients of a SDM model to estimate the spatial spillover effect. Therefore, we employed the method of decomposition to obtain the direct effect, indirect effect and the total effect of each influencing factor of EIWB. Moreover, ECPC and WB were also used as explained variables in the decomposition of spatial effects to further explore if the factors have effects on EIWB through influencing energy consumption or residents’ health. Due to limited space, the direct effect, the indirect effect and the total effect of each explanatory variable in the spatial Durbin model based on spatial contiguity matrix are shown in Table 6.

It can be seen from the measurement results in Table 6:

Industrial structure. Industrial structure had a positive direct effect and indirect effect on EIWB, which indicates the rise of industrial proportion of a region will increase EIWB in not only the local area but also neighboring areas. (1) The indirect effect of industrial structure on EIWB is mainly due to the damage caused by industrial enterprises to residents’ health in neighboring areas. The results in Table 6 show that its indirect effect on ECPC was not significant while its indirect effect on WB was significantly negative. It indicates industrial enterprises might cause the problem of ‘cross-border pollution’ [60] and does harm to the health condition of residents in neighboring areas, which accounts for the positive indirect effect of industrial structure on EIWB. (2) The significant positive direct effect of industrial structure on the EIWB was consistent with expectations. According to previous studies, on the one hand, industrial enterprises are mostly energy-intensive and regions with higher levels of industrialization consume more energy [61]. On the other hand, due to the release of industrial pollutants, residents’ health and well-being will also be damaged [62].

Urbanization level. The indirect effect of this variable does not show that there is a spatial spillover effect on the EIWB of urbanization, and the positive direct effect shows that the increase of urbanization level would also lead to the rise of EIWB in the local region. It can be seen in Table 6 that urbanization had significant direct effects on ECPC and WB, which confirmed the fact that urban residents consume more energy and enjoy better health and well-being than rural residents. High energy consumption led to an increase in EIWB while good health condition led to a decrease in EIWB. Therefore, the positive direct effect on EIWB shows that urbanization had a stronger effect on energy consumption than its effect on the improvement of residents’ health. The conclusion was consistent with the conclusions obtained by scholars such as Yan [38], Sheng and Guo [63] from the perspective of energy efficiency.

Energy structure. The direct effect of the energy structure was significantly positive at the level of 1%, and the indirect effect was significantly negative at the level of 5%. Since the direct effect and the indirect effect were opposite, the total effect was not significant. (1) The indirect effect of energy structure was negative, which means that the increase of coal’s share of energy consumption in the region could reduce the EIWB in neighboring regions. One possible explanation for this is pollution transfer [64]. The region uses coal to generate electricity and then sends electricity to its neighboring areas. Since electricity is an efficient and clean energy to the neighboring areas, it will result in more efficient energy use and less pollution in the neighboring areas. Energy structure’s significant negative indirect effect on ECPC and positive indirect effect on WB in Table 6 can support this view. (2) Coal combustion often produces a variety of toxic and harmful substances including carbon monoxide, sulfur dioxide and nitrogen oxides, so the direct effect of energy structure on local EIWB was significantly positive.

Foreign direct investment. Both direct and indirect effects of FDI were negative at the level of 1%. According to the results, every 1% increase in FDI will reduce the EIWB in local region by 0.083% and the EIWB in the neighboring region by 0.287%. This result verified the establishment of the “Pollution Halo” hypothesis in the context of China and indicates that the advanced management experience and emission reduction technology brought by FDI was conducive to the improvement of environment. It can be found in Table 6 that the introduction of FDI could not decrease the energy consumption in local and neighboring areas because the direct and indirect effects of FDI on ECPC were both significant positive. However, FDI also had positive direct and indirect effects on WB. The spatial spillover effect of FDI on reducing EIWB illustrates its impact on improving residents’ health was greater than its impact on increasing energy consumption.

Per capita disposable income. The indirect effect of per capita disposable income was not significant, but the direct effect was positive. The positive direct effect on ECPC suggested higher income groups consume more energy, and the positive direct effect on WB suggested their health and well-being were better than that of lower income groups. Like the impact of urbanization on EIWB, the increase in energy consumption caused by higher incomes was more obvious than the improvement in health and well-being. In Section 3.1, we discussed the difference between the energy intensity and the EIWB in Tianjin. We found that the economic output (presented by GDP) in Tianjin showed a clear upward trend, but the residents’ health and well-being did not show the same trend. On the contrary, the level of health and well-being in many years had declined from the previous year. Tianjin is a typical high-income region, and per capita disposable income is also a form of economic output. In fact, per capita disposable income has a high correlation with GDP. In other words, the results show the shortcomings of measuring energy efficiency only by economic output again: increasing economic output does not mean that the residents’ health and well-being can also improve. Therefore, it is necessary to re-examine the energy efficiency using EIWB.

Public health expenditure. At the significant level of 1%, both direct and indirect effects of public health expenditure were negative, which indicates that increased public health expenditure could reduce EIWB not only in the local region but also in neighboring regions. The spatial spillover effect of public health expenditure on reducing EIWB results from its impact on improving residents’ health in neighboring regions. As is shown in Table 6, the indirect effect of public health expenditure on ECPC was not significant while the indirect effect on WB was significantly positive. A possible explanation is that residents in neighboring areas can go to regions with more advanced medical technologies in order to obtain better treatment. Thus, the negative indirect effect of public health expenditure reflects the fact that residents in neighboring areas can also benefit from local medical resources [65].

Technological progress. Both the direct and indirect effects of technological progress were negative at the significant level of 1%, which indicates it has a spatial spillover effect on the reduction of EIWB. The results in Table 6 also show that technological progress had significant effects on ECPC and WB and its impact on improvement of residents’ health was greater than that on increasing energy consumption. It confirms the inference in other studies that technological progress had positive spillover effects on energy efficiency, reduction in industrial pollutant emissions and improvement of ecological environment [66,67]. These positive externalities brought by technological progress are reasons why technological progress can reduce the EIWB in neighboring areas.

## 4. Conclusions and Policy Implications

### 4.1. Conclusions

In this paper, the panel data of 30 provinces in China from 2005 to 2016 were used as research samples to calculate EIWB, and the spatial distribution characteristics of EIWB and the reasons for high EIWB in some regions. In addition, this paper also establishes the corresponding space Durbin model on STIRPAT model, and investigates the spatial spillover effect of various influencing factors of EIWB. The main conclusions are as follows:

In terms of the spatial distribution, the EIWB in most provinces showed low (L–L) agglomeration. The provinces with high EIWB were concentrated in the northern regions of China. The per capita energy consumption in the provinces with high EIWB was generally higher than the national average, while the health and well-being was significantly lower than the national average. In particular, the study also found that the increase in the EIWB in Tianjin was due to the fact that the per capita energy consumption in the region had not significantly improved residents’ health and well-being.

In terms of influencing factors of EIWB, (1) Industrial structure and energy structure had positive effects on EIWB in local area, because the rise of industrial proportion and coal consumption will increase energy consumption and damage residents’ health. In addition, industrial structure had a spatial spillover effect. It can result in an increase of EIWB in neighboring areas due to the cross-border pollution caused by industrial enterprises. (2) The rise of the urbanization level and per capita disposable income will increase EIWB in local area because its impact on promoting energy consumption is greater than its impact on improving residents’ health. (3) Public health expenditure, FDI and technological progress could significantly reduce the EIWB in local and neighboring areas. Public health expenditure reduced EIWB mainly through improving residents’ health and it had no significant impact on energy consumption. Different from public health expenditure, FDI and technological progress had a significant positive impact on energy consumption. However, they had a more significant impact on improving environment and resident’s health.

### 4.2. Policy Implications

Some policy implications could be provided to the policy makers according to our findings:

(1) Through the case of Tianjin, this study proved that the decrease of EI could not simply equal to the increase of residents’ health and well-being. Therefore, the government could consider using EIWB as a reference for the formulation of energy policies to ensure that energy consumption was conducive to the improvement of residents’ health and well-being.

(2) It was found that the rise of industrial proportion and coal consumption would promote energy consumption as well as damage residents’ health. From the perspective of energy consumption, local governments in areas with industry and large coal consumption should make plans to transfer its mode of economic development and develop industry with low energy consumption. Meanwhile, the governments should also fund scientific institutions to look for alternative energy and efficient use of coal. From the perspective of residents’ health, pollution detection and warning mechanism should be set up in areas with intensive industry and large coal consumption. The government can carry out real-time detection of the pollutant content in the air and water, and inform the residents of time and area when the pollutant exceeds the standard through social media such as Microblog (China), WeChat (China), Twitter and Facebook to reduce the possibility of residents’ exposure to pollutants and improve residents’ health. Moreover, industrial enterprises might cause cross-border pollution and do harm to neighboring residents’ health. Thus, the government should develop strict pollutant discharge standards and apply environmental regulations such as emission trading and Pigouvian taxation to reduce the threat of industrial pollutant to residents’ health.

(3) The empirical results confirmed that although urban residents and high-income people have better health condition, they also consume large amount of energy, which leads to the increase of EIWB. Therefore, it is necessary to lower their energy consumption through guiding them to implement energy-saving behavior. Given that this kind of people are usually insensitive to the price of energy while they have significant psychological needs for social identity. The government can put energy-saving slogans and energy-saving public service advertisements in places where such people often appear, such as shopping malls, hotels and high-end residential areas. These kinds of propaganda can be used to make them realize that the implementation of energy-saving behavior can get social recognition, while the waste of energy will reduce social recognition, and then urge them to take the initiative in energy-saving behavior. Furthermore, the government can make use of the demonstration effect of high-income people’s energy-saving behavior to form a good atmosphere of energy conservation in the whole society.

(4) The empirical results show that although public health expenditure had no significant impact on energy consumption, it would reduce EIWB through improving local and neighboring residents’ health. Therefore, local governments should increase public health expenditure and fund corresponding medical and health projects for environmental problems caused by energy consumption. For example, in areas with severe haze, the government can subsidize medical institutions to issue free protective masks during the period of severe air pollution, and add special protection provisions for respiratory diseases in the medical insurance to reduce the medical cost of residents’ prevention and respiratory disease treatment. It is also suggested that the government should upgrade and increase the delivery of medical equipment for the treatment of respiratory diseases. In order to enhance the impact of public health expenditure on improving neighboring residents’ health, the government should encourage the share of medical resource through building medical malls and providing online inquiry service. Through these measures, the economic welfare generated by energy consumption can be more used to improve residents’ well-being, and then improve the energy efficiency from the perspective of health.

(5) It is also suggested that the government should introduce FDI and encourage technological progress. Even if it was proved in the study that FDI and technological progress would raise energy consumption, implementing the above policies could reduce EIWB through improving environment and residents’ health in local and neighboring regions. However, due to the positive externality caused by spatial spillover effects, local governments may not be effectively encouraged. In this regard, the central government must set up a comprehensive and reasonable evaluation mechanism, correctly evaluate all the contributions made by a region in reducing the EIWB, and reward them accordingly to properly motivate governments. At the same time, a cross-regional cooperation mechanism needs to be established to provide a platform for consultation and cooperation between different administrative regions in the overall planning and coordination of their respective functions to reduce the EIWB, so as to eventually promote coordinated governance and common improvement between regions.

## 5. Limitations and Future Research

In this study, several indicators that could be used to describe the health status and well-being of residents were considered in the measurement of EIWB. However, due to the absence of provincial-level data of some indicators (such as average life expectancy and disability adjusted life, etc.), we could not include them in the measurement of EIWB. The absence of data also limited our further discussion on this issue, such as how EIWB at the municipal level changes. In addition, due to the untimely update of some data, this study failed to analyze EIWB after 2016. Future research should try to obtain more detailed data to achieve in-depth study of EIWB.

## Figures and Tables

**Table 1:** Research on total factor energy efficiency.

Author	Input and Output Variables	Method
Sueyoshi et al. [20]	GPR, CO_2_, SO_2_, dust, waste water, ammonia nitrogen, energy, labor, capital	DEA, Malmquist productivity index
Apergis et al. [21]	productive capital stock, labor, renewable energy, non-renewable energy	SBM
Feng et al. [22]	energy, labor, capital stock, number of patent authorizations, GDP	SBM, Tobit model
Zhao et al. [23]	energy, labor, capital stock, GDP	SFA
Long et al. [24]	GRP, SO_2_, capital, labor, coal	Directional distance function
Yang et al. [25]	labor, capital stock, energy consumption	DEA, Tobit model
Li et al. [26]	capital, labor, industrial energy consumption, industrial output, CO_2_	DEA

Note: DEA represents ‘Data Envelopment Analysis’; SBM represents ‘Slack Based Model’; SFA represents ‘Stochastic Frontier Analysis’.

**Table 2:** Wald, likelihood ratio (LR) and Hausman test results.

Test	Statistics	*p*-Value
Wald spatial error	31.95 ***	<0.001
Wald spatial lag	34.90 ***	<0.001
LR spatial error	250.27 ***	<0.001
LR spatial lag	198.04 ***	<0.001
Hausman	17.80 **	0.013

Note: *** and ** respectively represent significant at the levels of 1% and 5%.

**Table 3:** Global Moran’s I of China’s EIWB.

Year	Moran’s I	E(I)	Sd(I)	z	*p*-Value
2005	0.408 ***	−0.034	0.119	3.726	<0.001
2006	0.394 ***	−0.034	0.119	3.607	<0.001
2007	0.375 ***	−0.034	0.118	3.462	<0.001
2008	0.385 ***	−0.034	0.120	3.504	<0.001
2009	0.354 ***	−0.034	0.117	3.336	<0.001
2010	0.396 ***	−0.034	0.116	3.703	<0.001
2011	0.381 ***	−0.034	0.118	3.535	<0.001
2012	0.367 ***	−0.034	0.117	3.416	<0.001
2013	0.409 ***	−0.034	0.115	3.861	<0.001
2014	0.405 ***	−0.034	0.116	3.797	<0.001
2015	0.358 ***	−0.034	0.115	3.406	<0.001
2016	0.355 ***	−0.034	0.115	3.376	<0.001

Note: *** indicates significant levels at 1%.

**Table 4:** Local agglomeration of China’s EIWB in 2006, 2011 and 2016.

Year	H-H	L-H	L-L	H-L
2006	Hebei, Shanxi, Inner Mongolia, Liaoning, Gansu, Qinghai, Ningxia, Xinjiang	Jilin, Heilongjiang, Sichuan, Shannxi	Beijing, Tianjin, Shanghai, Jiangsu, Zhejiang, Anhui, Fujian, Jiangxi, Shandong, Henan, Hubei, Hunan, Guangdong, Guangxi, Hainan, Chongqing, Yunnan	Guizhou
2011	Shanxi, Inner Mongolia, Liaoning, Jilin, Gansu, Qinghai, Ningxia, Xinjiang	Beijing, Hebei, Heilongjiang, Sichuan, Shannxi	Shanghai, Jiangsu, Zhejiang, Anhui, Fujian, Jiangxi, Shandong, Henan, Hubei, Hunan, Guangdong, Guangxi, Hainan, Chongqing, Guizhou, Yunnan	Tianjin
2016	Shanxi, Inner Mongolia, Liaoning, Qinghai, Ningxia, Xinjiang	Beijing, Hebei, Jilin, Heilongjiang, Sichuan, Shannxi, Gansu	Shanghai, Jiangsu, Zhejiang, Anhui, Fujian, Jiangxi, Shandong, Henan, Hubei, Hunan, Guangdong, Guangxi, Hainan, Chongqing, Guizhou, Yunnan	Tianjin

**Table 5:** Estimation results of the impact of influencing factors on EIWB.

Variable	*W*_1_		*W*_2_		*W*_3_	
	Coefficient	*p*-Value	Coefficient	*p*-Value	Coefficient	*p*-Value
lnis	0.590 ***	<0.001	0.532 ***	<0.001	0.964 ***	<0.001
lnul	1.152 ***	<0.001	1.190 ***	<0.001	1.920 ***	<0.001
lnes	0.424 ***	<0.001	0.426 ***	<0.001	0.161 *	0.093
lnfdi	−0.080 ***	0.002	−0.159 ***	<0.001	−0.306 ***	<0.001
lnpcdi	0.835 ***	<0.001	0.378 *	0.080	−0.138	0.445
lnhe	−0.198 ***	0.009	−0.029	0.772	0.131	0.231
lnt	−0.146 ***	<0.001	−0.089 **	0.038	−0.264 ***	<0.001
W*lnis	0.841 ***	0.001	−0.393	0.276	0.743 *	0.074
W*lnul	−0.088	0.808	0.750	0.108	1.522 ***	0.008
W*lnes	−0.336 **	0.021	0.832 ***	0.001	−0.459 **	0.013
W*lnfdi	−0.266 ***	<0.001	−0.404 ***	<0.001	−0.116	0.142
W*lnpcdi	−0.292	0.333	0.537	0.284	0.464	0.305
W*lnhe	−0.717 ***	<0.001	0.436	0.114	−0.623 *	0.010
W*lnt	−0.369 ***	<0.001	−0.222 *	0.070	0.628 ***	<0.001
*ρ*	0.599 **	0.042	0.184 **	0.047	0.064 **	0.047
Sigma2_e	0.110 ***	<0.001	0.165 ***	<0.001	0.203 ***	<0.001

Note: ***, ** and * indicate significant levels at 1%, 5% and 10%, respectively.

**Table 6:** Direct, indirect and total effects of the spatial Durbin model.

Variable	EIWB			ECPC			WB		
	Direct Effects	Indirect Effects	Total Effects	Direct Effects	Indirect Effects	Total Effects	Direct Effects	Indirect Effects	Total Effects
IS	0.607 *** (5.67)	0.924 *** (3.28)	1.531 *** (4.53)	0.385 *** (4.55)	0.131 (0.51)	0.516 * (1.66)	−0.070 ** (−2.18)	−0.174 ** (−1.99)	−0.243 ** (−2.35)
UL	1.142 *** (6.16)	0.006 (0.02)	1.147 *** (3.81)	0.788 *** (10.86)	−0.339 (−0.18)	0.449 ** (2.45)	0.231 *** (7.79)	−0.180 (−0.27)	0.051 (0.83)
ES	0.425 *** (6.20)	−0.331 ** (−2.24)	0.094 (0.70)	1.351 *** (8.12)	−0.992 ** (−2.20)	0.358 (0.78)	−0.026 (−0.38)	0.364 ** (2.28)	0.338 ** (2.16)
FDI	−0.083 *** (−3.32)	−0.287 *** (−4.97)	−0.370 *** (−5.53)	0.181 *** (8.74)	0.132 *** (4.80)	0.313 *** (7.09)	0.269 *** (2.70)	0.503 ** (2.18)	0.772 ** (2.35)
PCDI	0.845 *** (5.17)	−0.260 (−0.82)	0.585 ** (2.21)	0.176 *** (2.69)	0.062 ** (2.36)	0.238 *** (3.13)	0.101 *** (3.93)	−0.012 (−1.32)	0.089 *** (3.55)
HE	−0.189 *** (−2.61)	−0.739 *** (−4.00)	−0.928 *** (−2.60)	0.558 (1.50)	0.075 (0.94)	0.131 (1.20)	1.090 *** (4.40)	0.098 (1.06)	1.188 *** (3.20)
T	−0.152 *** (−4.36)	−0.394 *** (−5.68)	−0.546 *** (−7.37)	0.360 *** (4.77)	0.620 *** (4.12)	0.980 *** (5.34)	0.463 ** (2.36)	1.034 ** (2.36)	1.497 *** (2.77)

Note: ***, ** and * indicate significant levels at 1%, 5% and 10%, respectively. The numbers in brackets are *t* statistic values.
